# Monitoring Indoor People Presence in Buildings Using Low-Cost Infrared Sensor Array in Doorways

**DOI:** 10.3390/s21124062

**Published:** 2021-06-12

**Authors:** Cristian Perra, Amit Kumar, Michele Losito, Paolo Pirino, Milad Moradpour, Gianluca Gatto

**Affiliations:** Department of Electrical and Electronic Engineering, UdR CNIT, University of Cagliari, Via Marengo 2, 09123 Cagliari, Italy; amit.kumar@unica.it (A.K.); michele.losito@unica.it (M.L.); paolo.pirino@unica.it (P.P.); moradpour@sdu.dk (M.M.); gatto@unica.it (G.G.)

**Keywords:** people counter, pattern recognition, IR sensor array, Z-Wave

## Abstract

We propose a device for monitoring the number of people who are physically present inside indoor environments. The device performs local processing of infrared array sensor data detecting people’s direction, which allows monitoring users’ occupancy in any space of the building and also respects people privacy. The device implements a novel real-time pattern recognition algorithm for processing data sensed by a low-cost infrared (IR) array sensor. The computed information is transferred through a Z-Wave network. On-field evaluation of the algorithm has been conducted by placing the device on top of doorways in offices and laboratory rooms. To evaluate the performance of the algorithm in varying ambient temperatures, two groups of stress tests have been designed and performed. These tests established the detection limits linked to the difference between the average ambient temperature and perturbation. For an in-depth analysis of the accuracy of the algorithm, synthetic data have been generated considering temperature ranges typical of a residential environment, different human walking speeds (normal, brisk, running), and distance between the person and the sensor (1.5 m, 5 m, 7.5 m). The algorithm performed with high accuracy for routine human passage detection through a doorway, considering indoor ambient conditions of 21–30 °C.

## 1. Introduction

Human monitoring has always drawn attention in many applications, including smart building. Determining the number of people inside each room in a building is a very important task for controlling that the safety norms imposed by laws are properly followed and that health system recommendations are fulfilled. This becomes more important in the context of the coronavirus pandemic, wherein it is essential to monitor people’s occupancy, especially in an indoor environment. In this context, designing a low-cost architecture for monitoring the number of people occupying rooms is a challenging task.

A building’s related information is often managed with so-called Building Information Modeling (BIM), a standard procedure employed for optimizing the planning and implementation of a system with the help of a software platform [1]. It requires the fulfilment of some key parameters that include building elements and equipment to be modelled, high-quality documentation, and necessary attributes for monitoring and interpreting available information [2].

The management of the public building, from the energy point of view, implies the knowledge of how and when the internal workspaces are used, and therefore by the instantaneous measurement of the number of people inside it. Thus, suitably training a neural network or using appropriate machine learning algorithms [3] to update a behavioural model of the building can allow rationalizing both the use of spaces and consequently the use of the electrical/thermal energy necessary for their lighting and air conditioning. In this scenario, sensor technology manifests as a powerful tool for acquiring data from a building that forms an important basis for BIM application.

Sensor technology started from the simple thermistor to advanced differential pyroelectric devices. Based on their operating principle, sensors are classified as mechanical and electronic, while in terms of communication and storage, they are classified as wired, wireless, and portable. In building applications, sensor technologies are used to monitor parameters such as temperature, humidity, CO_2_, lighting, and occupancy. Implementation challenges of sensors network can vary notably for different building applications and depends on factors such as building size, location, number of devices, and preferred technologies [4].

A huge range of communication and wireless networking methods, such as Zigbee, Bluetooth Wi-Fi, Z-Wave, etc., are available in the market to complement the proper functioning of a sensor system inside a building. Hayat et al. in [4] have provided a detailed overview of prominent wireless technologies employed in buildings. Kim et al. in [5] used the Zigbee protocol to implement a hybrid wireless sensor network in the building set-up. In a recent study [6], the authors investigated the application of a smart sensing network embedded with passive infrared (PIR) CO_2_ sensors using BACnet communication protocol and noted a reduction of building-operational cost and energy. Yassein et al. in [7] utilised the Z-Wave protocol and discussed its application in smart homes.

Numerous technologies are proposed in the literature for human presence detection [6] that exhibit different features such as costs, resolution, privacy, etc. For instance, conventional video camera systems used for surveillance are accurate but expensive and require significant infrastructure to process pieces of information to extract relevant features and are intrusive. Several low-cost sensor systems have been utilised to preserve privacy but with a low-resolution sensing feature. In literature, several studies have proposed a combination of different sensing modalities and machine learning approaches for counting and identifying people inside buildings, utilising a low-cost sensor set-up.

Moreover, recent research has shown that surveillance video camera data can be analysed in order to extract information relating to suspicious human behaviour and identity [8,9]. In particular, gait recognition is an emerging biometric technology that identifies people through the analysis of the way they walk [10].

Sun et al. in [11] used a low-cost pyroelectric sensing system for human-monitoring applications, and the sensor array was built using single PIR sensors. Yang et al., in [12], used a PIR sensor mesh network, and an improved Kalman filter and a particle filter are used for human tracking. Hao et al., in [13], investigated a low-cost alternative to infrared video sensors, i.e., a PIR sensor system for tracking and identifying people based on their body heat radiation using an Expectation-Maximization-Bayesian tracking scheme. Chowdhury et al. in [14] investigated a low-cost security system using a small PIR sensor built around a microcontroller by sensing thermal perturbations with the surrounding environment due to the presence of individuals. Caidedo et al., in [15], proposed an ultrasonic sensor array prototype for reliable indoor human presence detection. Unlike pyroelectric, thermopile sensors can detect both stationary and motion activities without compromising privacy.

In this work, a thermal array (64 thermopile sensing elements, 8 × 8 grid) IR sensor was set-up to count the number of people inside a room using a novel algorithm for automated recognition of patterns in data (pattern recognition). In Table 1, we confine our attention to research works that have utilised the same (8 × 8 pixels) thermal array IR sensors to monitor human movement/presence but employed different computational approaches to analyse the data.

Basu and Rowe, in [16], developed a low-cost method to estimate the direction of human motion (with 80% accuracy) using a 64 pixels IR sensor array and using a support vector machine algorithm. Mohammadmoradi et al., in [17], employed a threshold-based technique (Otsu’s binarization) to estimate the people flow (with an average 93% accuracy) through doorways using a low-resolution IR sensor array.

Trofimova et al., in [18], utilised the noise removal technique based on the Kalman filter to detect human presence in an indoor environment using 8 × 8 pixels thermal sensor arrays. Jeong and co-workers, in [19], proposed a probabilistic method with image pre-processing and post-processing techniques for human detection using heat signatures from a low-resolution thermal IR sensor array system.

Qu et al., in [20], proposed a thermopile sensor array deployed horizontally with a height of 3.5 m for indoor localization and a human tracking system with a Kalman filter. The designed adaptive threshold technique to preserve human targets and remove background by the authors functions well for indoor multiple human targets. Within a smart building set-up, Maaspuro, in [21], studied an application of IR sensor array as a doorway occupancy counter using Kalman filter tracking algorithm and reported an accuracy between 89–92%.

Doherty et al., in [22], implemented a novel indoor localization system by combining sensor technology and machine learning methods (logistic regression, K-Nearest neighbours, support vector machine, and a feedforward neural network) to collect and analyze human occupancy data in an indoor toilet. They reported an accuracy value between 98–99%.

For completeness, we also report studies that have employed better resolution IR sensor arrays for indoor human tracking. Spasov et al., in [23], proposed the use of a 32 × 24 pixels thermal detector with an open-source hardware board for developing a solution with applications for monitoring of the human presence and home appliances. Gu et al., in [24], proposed a dynamic fuzzy spatiotemporal background removal technique for human activity perception based on a 32 × 24 pixels thermopile sensor array (MLX90640, Melexis Corporation, Belgium).

The goal of our study is to validate the functionality of the proposed pattern recognition algorithm that allows determining the number of people in a room, using a low-cost and easy to install sensor system on top of doorways. The total number of people in the building can be then calculated by adding up this information. To do so, we designed and developed a low-cost smart IR sensor system to track the number of people entering and exiting the environment, which required constant monitoring. In detail, we present the design, construction, and programming of a Z-Wave-based sensors. The development board used is called Z-Uno. It is a board for developers that allows creating a customized Z-Wave device, which then implements the Z-Wave protocol management utilising the pre-installed software. Further, we studied a pattern recognition algorithm for analysing the IR sensor time-series data and identifying events of people crossing the doorway. The main requirements of this algorithm are low computational complexity and low memory footprint. The low memory footprint is achieved by using reference data in the last five sensor readings. The developed algorithm was then implemented into the microcontroller present in the sensor device. Finally, the algorithm’s accuracy to detect different human walking speeds at temperature ranges typical of the residential environment was investigated by performing various stress tests on synthetic data sets simulating temperature ranges typical of a residential environment, different human walking speed (normal, brisk, running), and different distances between the person and the sensor (1.5 m, 5 m, 7.5 m).

In summary, the main contributions of this work are:

(1) Study and development of a low-computational complexity and low memory footprint pattern recognition algorithm.
(2) Development of a low-cost people-counting device with a microcontroller implementing the proposed algorithm.

The paper is organized as follows. In Section 2, we describe the proposed architecture. In Section 3, we present the proposed pattern recognition algorithm. In Section 4, we present the experimental analysis scheme. In Section 5, we discuss the three phases: data acquisition and calibration of algorithm, validation, and simulation of the real-time human-passage experiment. Finally, the conclusions and future work are described in Section 6.

## 2. Proposed Architecture

The proposed architecture is composed of three main components: a Panasonic Grid-EYE 64 pixel IR sensor array, a Z-Wave network, and a signal-processing module [25]. The infrared sensor captures the thermal image of the user’s passage, the I2C protocol passes the signal to the processing module, which implements the proposed pattern recognition algorithm, and after that, the data are sent via Z-Wave protocol to the network. These components are discussed in the following section.

**Figure 1:**
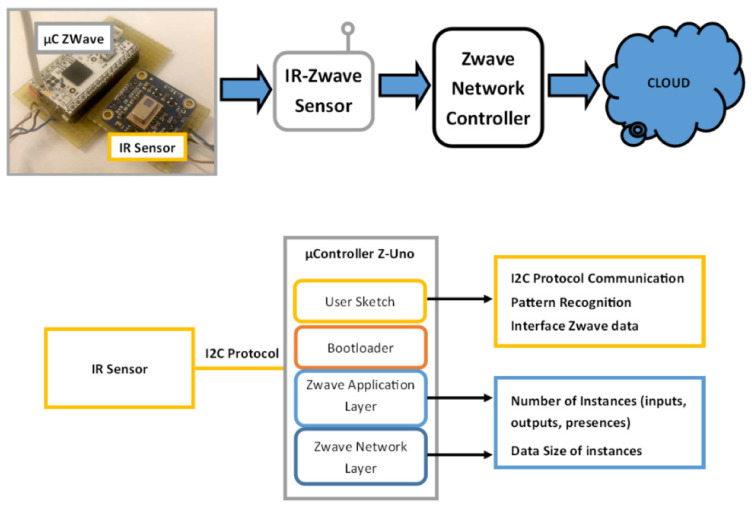
A schematic representation of the proposed architecture.

A schematic representation of the proposed architecture is shown in Figure 1.

### 2.1. Infrared Sensor

A GridEye sensor was used for the development and test of the proposed architecture. A GridEye sensor is a thermopile array device consisting of a matrix of 8 × 8 IR sensors, which are connected to the analog-to-digital converter (ADC) and internal memory, all managed by an integrated microcontroller that provides a digital interface via I2C protocol (serial communication protocol). The approximate cost of the sensor used in this paper is ~$50.

When considering the cost-effectiveness of indoor human-counting solutions, it is preferable to use low-resolution (8 × 8 pixels) thermal IR sensors instead of expensive high-end thermal cameras, which could still cost up to thousands of dollars.

The device is constituted by an 8 × 8 array of IR sensors (64 pixels), which performs the acquisition of comprehensive temperature data, thus obtaining thermal images and temperature gradients. The sensitivity of an IR sensor derives from the photometric sensitivity of each detector at each pixel. An IR sensor has multiple pixels that all work together to create a complete view of the area. By its nature, it protects the host environment’s privacy, contrary to what is possible with conventional cameras.

**Figure 2:**
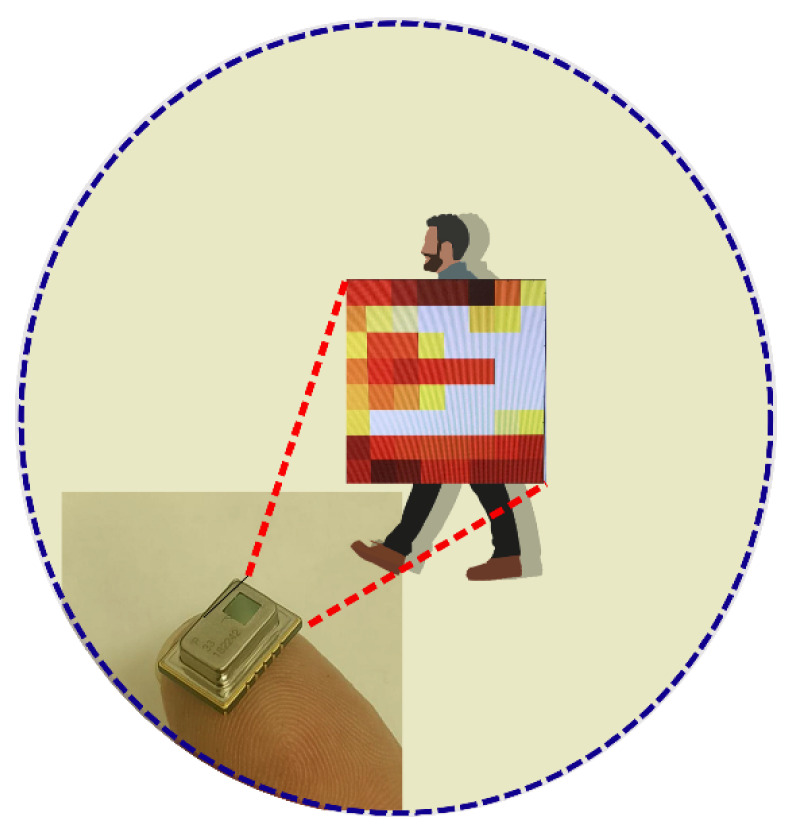
Infrared sensor for tracking human passage movement.

The sensor capturing a normal human passage movement is represented in Figure 2.

The chip integrates within its interior a lens that supports a viewing angle of 60° and an I2C visual interface that allows obtaining measurements with a frame rate from 1 to 10 frames per second. The device offers an interrupt pin for applications that require event management “critical-time,” a PIN for the selection of 2 I2C addresses. In Table 2, the essential features of the Grid-Eye sensors set-up are shown.

### 2.2. Z-Wave Installed IR Sensor

The IR devices were connected using the Z-Wave wireless communication protocol (based on mesh network topology). Z-Wave is a low-power wireless technology, easy to implement inside buildings, and requires low maintenance costs. Through the three values, the Z-Wave sensor network offers the treatment as three distinct Z-Wave channels containing, respectively: (i) the total number of inputs, (ii) the total number of outputs, and (iii) the number of people currently present within the environment. These values can be updated every 60 s, depending on the Z-Wave network configuration or need for ambient monitoring.

The tracking of human passage is performed by acquiring frames from the IR sensor and processing them through a pattern recognition algorithm, implemented in C++ language, that is executed cyclically inside the microcontroller of the Z-Uno board.

Through our developed algorithm, it was possible to perform the count of people entering and exiting the environment by querying the IR sensor device using the I2C protocol and analysing the time evolution of the captured frames. 

Different sequence patterns were obtained for the user’s movement in the vicinity of the sensor, for example, “input,” “fake entry,” “output,” and “fake exit,” as defined in Table 3. In this regard, in the following section, we present a detailed description of the proposed algorithm for people moving through a doorway.

## 3. Algorithm for Monitoring People Crossing a Doorway

### 3.1. Case Study and Requirements

**Figure 3:**
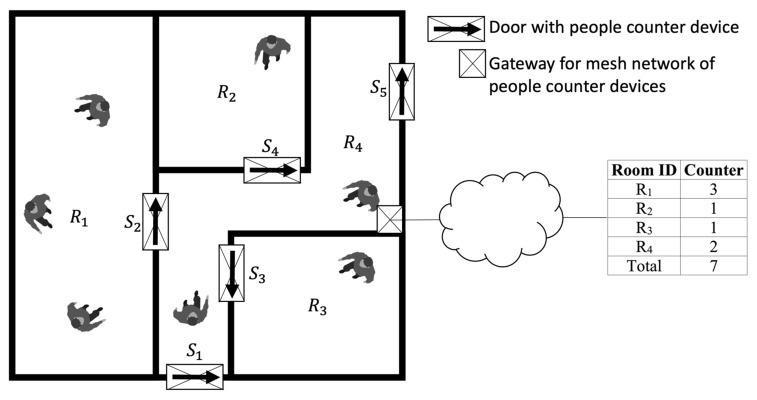
Case study: monitoring people presence in buildings.

The case study under consideration, which is termed indoor people counting, is represented in Figure 3. In this case study, those responsible for safety of people within the building needs to have exact information on the distribution of the people inside the building and, in particular, the number of people for each room/corridor and the total for the building. A small device is mounted on the top of the door casing. The device has a square infrared sensor facing the floor, aligned with the door casing, and implements a pattern recognition algorithm for locally processing the sensor data, and, in the case of activation, signals which event has occurred to a mesh network with the other devices in the building. The mesh network faces a gateway connected to a cloud service that collects, processes, and stores the relevant information.

The main requirements for the proposed algorithm are:

- Complexity, the proposed algorithm shall employ low computational complexity modules;
- Memory, the proposed algorithm shall target a low memory footprint;
- Accuracy, the proposed algorithm shall target full accuracy (100%) in stable indoor environmental conditions (22–26 °C) and high accuracy (higher than 95%) in variable indoor environmental conditions (18–32 °C).

### 3.2. Proposed Algorithm

The objective of the proposed pattern recognition algorithm is to analyse the last four frames in a sliding FIFO buffer captured by the infrared sensor to extract the information summarized in Table 3. On-field experiments revealed that some people might just stop at the doorway and then might finally enter the room or go away without completing the entrance movement. In order to correctly identify the action performed by the people, the hold state must be considered as one of the possible states. As discussed in Section 2.1, the sensor generates frames corresponding to 8 × 8-pixel images. The frame rate is between 1 and 10 frames per second.

**Figure 4:**
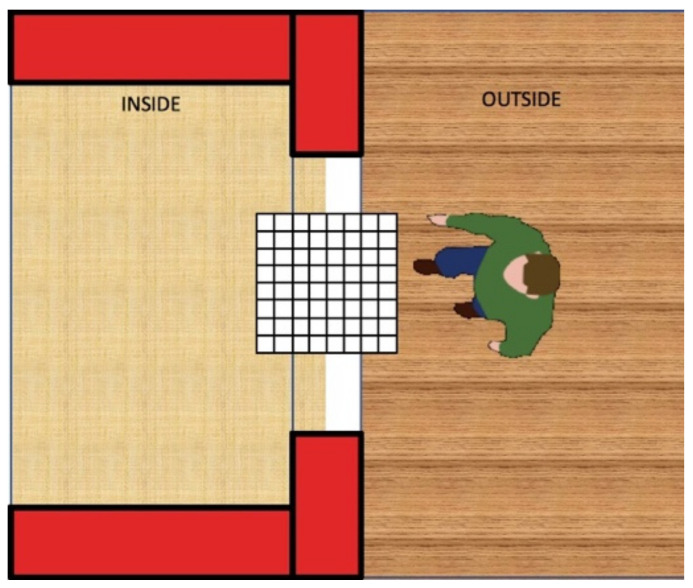
Reference scenario of an event: user’s passage.

The reference scenario considered for devising the pattern recognition algorithm is shown in Figure 4. There is an IR sensor with 8 × 8 elements (the sensor in Figure 4 is magnified to show its position in the scene) fixed on the top of the doorway. With reference to the location of the sensor in Figure 4, we define “inside” of the space as on the left of the sensor and “outside” of the space as on the right of the sensor. In the figure, a subject is outside, moving to go inside. Once the subject is inside, the algorithm should signal the entrance message (E).

While the user is moving through the doorway, different elements of the sensor are activated. Referring to Figure 4, if the user moves from the right to the left, the sensor should reflect this movement by enabling at first some sensing elements and the rightmost column of sensors (column 8) and then activating elements in the following columns (from column 7 to column 1).

Each 8 × 8 matrix (frame) captured by the sensor is stored in a first-in-first-out (FIFO) buffer. As a frame is processed by the proposed algorithm, it is removed from the buffer.

The method consists of partitioning the frame into four vertical stripes, computing a moving average threshold (Equation (4)) for identifying a possible passage of the subject in the area covered by one of the vertical stripes, and finally use the evolution of the features extracted from each stripe for inferring the most probable action taken by the subject according to the patterns described in Table 3. 

Let $F_{t}\left(x,\;y\right),\;x\in \left[1,8\right],\;y\in \left[1,8\right]$ denote the t-th frame captured by the sensor, and the corresponding equalized image is computed as

(1) $$I_{t}\left(x,y\right)=\frac{F_{t}\left(x,y\right)\;-min\left(F_{t}\right)}{max\left(F_{t}\right)-min\left(F\right)}$$

The image is then partitioned into four slices

(2) $$I_{t,i}=I_{t}\left(x,y\right)\;\mathrm{for}\;\left(i-1\right)*2+1\leq x\leq \left(i-1\right)*2+2,1\leq y\leq 8,i\in \left[1,4\right]$$

**Figure 5:**
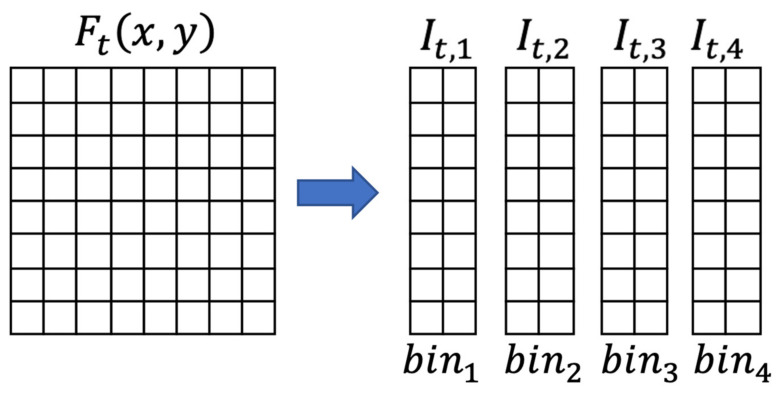
Partitioning a frame into bins.

Figure 5 shows the sensor elements associated to each bin.

From each slice (*i*), the power is computed as

(3) $$P_{t,i}=\frac{\sum I_{t,i}^{2}{}_{}}{16}$$

where 16 is the number of pixels (2 × 8) for each slice.

**Figure 6:**
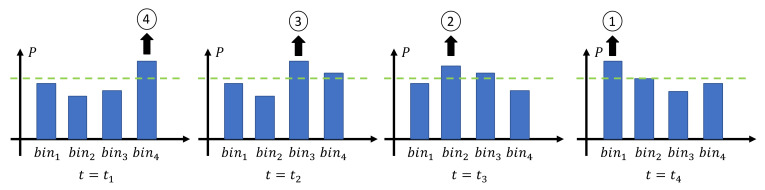
Examples of the bin’s time series when a subject is moving from the right to the left under the infrared sensor.

The threshold ($T_{t}$) for discriminating between no movement of subject crossing is computed as follows:

(4) $$T_{t}=k\sum_{i=1}^{4}\sum_{t^{\prime }=t-4}^{t}P_{t^{\prime },i}$$

where *k* is a normalizing coefficient. This coefficient has been chosen through a numerical analysis of the dataset, aiming at identifying a threshold *T* higher than the powers (Equation (4)) of each bin without activity; the obtained value was kept constant for all the subsequent studies. When the power of a bin is higher than the threshold, we assume that a subject is passing under that bin, and the corresponding bin number is produced as output. For example, referring to Figure 6, if the subject is moving from the right to the left, the output generated after processing frame $t_{4}$ is [1–4], meaning the following conditions have been verified: $P_{t_{1},4}>T_{t_{1}}$, $P_{t_{2},3}>T_{t_{2}},\;P_{t_{3},2}>T_{t_{3}}$, $P_{t_{4},1}>T_{t_{4}}$, with $t_{1}<t_{2}<t_{3}<t_{4}$. The time series of the bin levels described in this example are shown in Figure 6.

A timeout of *N* frames, chosen empirically (*n* = 40) during the experimental analysis from the last event detection (bin power higher than the threshold), is used for a periodic reset of the algorithm.

Finally, the bin time series are converted to the events in Table 3 according to the matching reported in Table 4. For example, the time series [1–4]] corresponds to an input event, while the times series [1–4]] corresponds to an output event. The hold state corresponds to time series values between 1 and 3 for a possible input event (IHO and IHI) or between 2 and 4 for a possible output event (OHI, OHO). The number of people in a room is computed as the difference between the input and output counters.

## 4. Experimental Analysis

The experimental set-up configured for assessing the performance of the proposed algorithm is described in this section. As shown in Figure 1, the system is composed of: (i) IR sensor (GridEye); (ii) mesh network (Z-Wave); and (iii) processing device (Z-Uno).

The pattern recognition algorithm was used for real-time processing of the frame sequences (thermal images) acquired from the sensor to identify the action performed by users: input (I), output (O), etc. An initial study was conducted to determine the actions normally performed by users and detected in the “IR region” and for computing the normalizing coefficient k (0.0163). To this end, the IR sensor was installed in the upper part of the access wall of the monitored environment, and the sensor was connected to a microcontroller that acquired the thermal images of a set of users. The users performed similar actions with approximately the same speed in a pre-defined sequence, reported in Table 5 and Table 6.

## 5. Results and Discussion

An 8 × 8 IR sensor array has 64 thermopile elements in an 8 × 8 grid format. The developed algorithm detects the passage of a person generating a numeric sequence (pattern) corresponding to the activated energy bins, as described in Section 3. For example, the complete passage from left to right generates a sequence of 012340. 

Depending on the generated numeric pattern, the algorithm transforms the initial value in “I,” “O,” and “H,” which corresponds, respectively, to “Input,” “Output,” and “Hold.” The algorithm performs the counting of the persons present within an environment exploiting the pattern recognition approach. 

The experimentation and validation for the IR sensor set-up were carried out in three phases. In the first phase, the acquisition of thermal images for the user’s passage was performed to obtain a set of offline calibration measures of the pattern recognition algorithm. The verification of the functionality of the pattern recognition algorithm in real-time and offline was performed in the second phase. Finally, the third phase concerned simulating a realistic human passage and assessing the algorithm in a normal environment. 

### 5.1. Acquisition of Thermal Images for Algorithm Calibration

**Figure 7:**
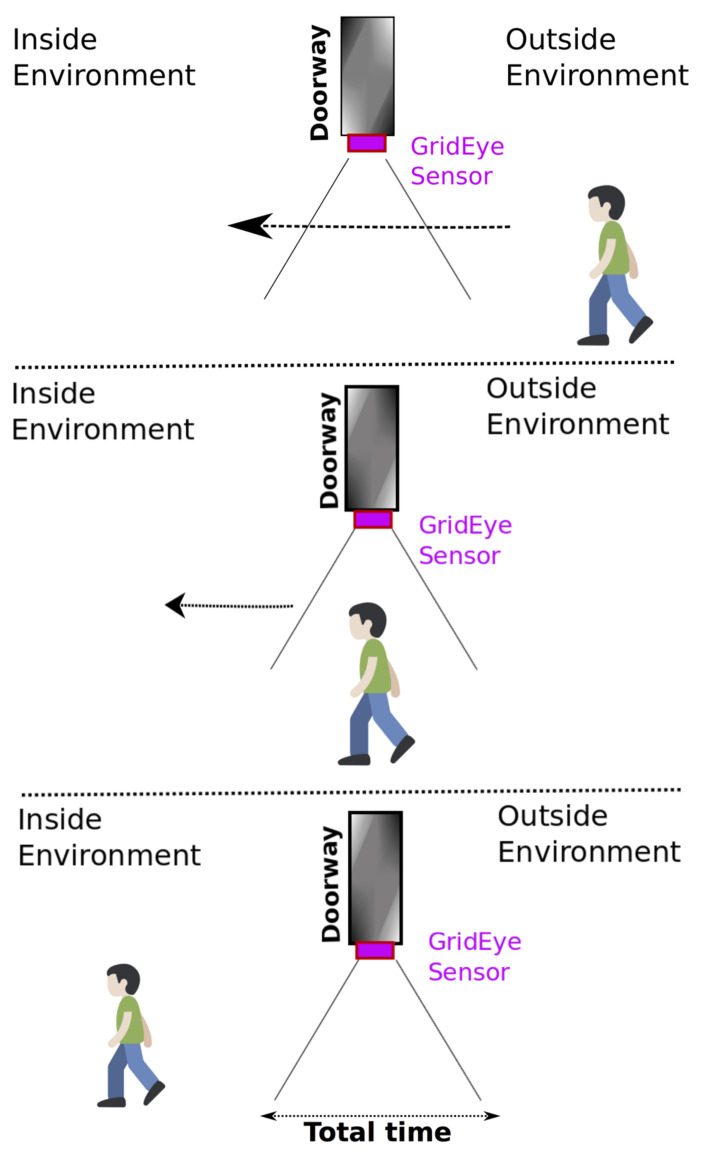
Depiction of a user crossing the doorway with the IR sensor set-up placed on the top.

In this phase, experimentation involved four individuals who were asked to repeat all the passages (Table 5) under the doorways to capture thermal images. For each experiment, the total time for all these passages (entering and exiting a room several times) has been recorded (Table 6). The sensor was installed at 2.30 m (m) in height, with the acquisition window facing downwards, and placed at the entrance of the environment in the crossbeam of the door opening (Figure 7).

The acquisition of frames was activated by using wireless control, and the measurements were saved to a micro-SD card in the text format file. The actions described in the pattern in Table 5 were performed by each user. Data processing was conducted on a personal computer to calibrate the offline algorithm parameters of pattern recognition. In Table 6, we report the height and duration of experimentation for each user. Each test involved the acquisition of the matrices, consisting of 8 × 8 values of temperature (where each matrix corresponds to one frame), with the maximum speed permitted by the microcontroller and saving them to a text file.

**Figure 8:**
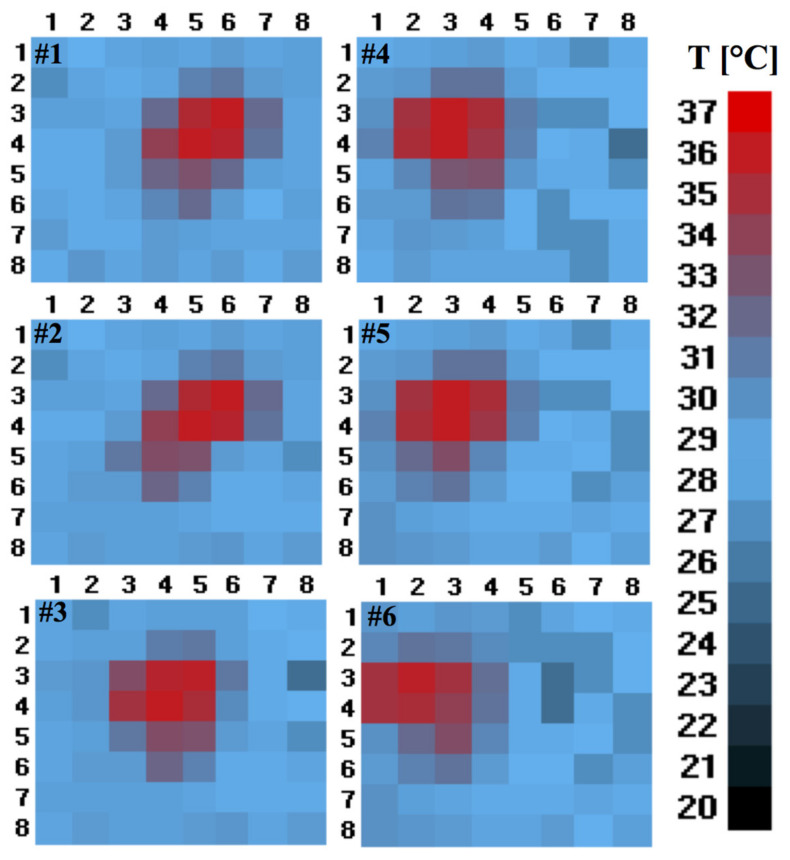
Six frames captured during the passage of a person recorded during the data collection and data processing of the software. The bar represents the temperature scale in °C.

In the detected thermal image shown in Figure 8, the coloured pattern represents the user’s entry/exit from the outside to inside, or vice versa, captured by the proposed system. 

### 5.2. Validation of Proper Functioning of the Developed Pattern Recognition Algorithm

The second phase involved the analysis of data collected during the measurements using Matlab (2018) and C++ programming codes. These implementations have been written relying on Matlab core functions and standard C++ libraries. The pattern recognition algorithm was developed, and its functionality was tested using the data collected from experiments. Subsequently, optimization of the algorithm parameters was conducted on the set of obtained measurements. The optimization consisted of the identification of minimum values for the size of the FIFO buffer and for the aperture of the timeout window allowing the correct recognition of the patterns. The developed software was then implemented into the microcontroller present in the proposed architecture. The software was compiled and loaded on the device to verify proper operation.

The device was tested for one month in our laboratory with stable environmental conditions (22–26 °C), reaching full accuracy (100%).

In order to further test the performance of the pattern recognition algorithm, synthetic frame sequences have been generated for simulating temperature ranges typical of a residential environment, different human walking speed (normal, brisk, running), and different distances between the person and the sensor (1.5 m, 5 m, and 7.5 m). Two categories of tests were performed: (i) using a step and (ii) using a ramp.

The perturbations with a temperature value of 37 °C were introduced into a part of the matrix elements (from right to left direction) of the 8 × 8 matrix, representing the ambient temperature as a base disturbance distribution. The two tests are described below.

*Step test.* The data collected in phase one was used to perform this test. The data consisted of a set of frames of 8 × 8 temperature values, representing different passages of a person below the sensor. The sequence of the actions are as follows: I, O, I, O, IHO, I, O, I, O, I, O, I, and OHI (see Table 3, for description).

**Figure 9:**
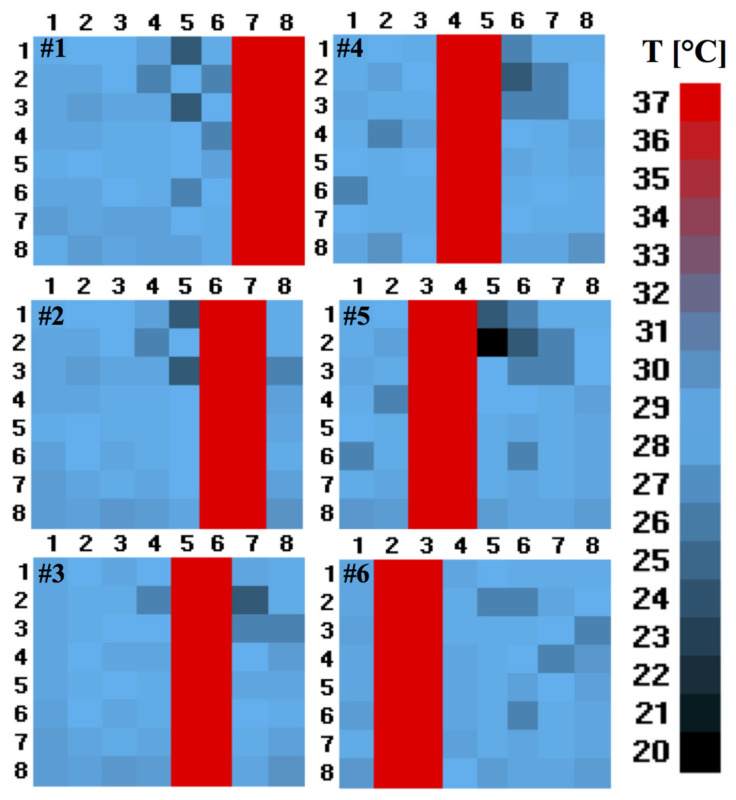
Representative illustration of “Step test” conducted with 6 frames. The step (perturbation) is shown in red, which scrolls from the right to left direction.

The objective of this test was to verify the algorithm’s response to the predetermined inputs set at different levels of ambient conditions and the perturbation of temperature. A step is a temperature perturbation that consists of an 8 × 1 temperature vector that slides in the right to left direction, which was then subsequently replaced by an 8 × 2 temperature matrix. For completeness, an image representing the perturbation movement (constituted by the 8 × 2 matrix) is shown in Figure 9.

The results obtained from the test highlighted the limits of the algorithm linked to the average values of the ambient temperature and the perturbation value. Precisely, with a temperature perturbation greater than 4 °C, the algorithm detects 100% of the events for ambient temperatures ranging from 21 to 31 °C. In contrast, no passage was detected with a perturbation value of less than 4 °C.

**Figure 10:**
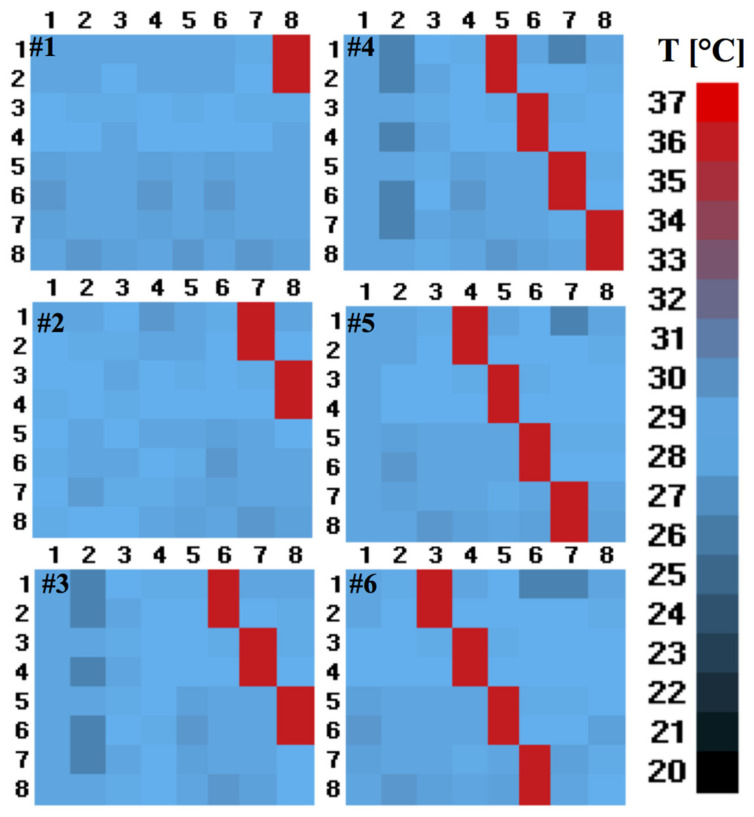
Representative illustration of Ramp test conducted with 6 frames. The red block represents perturbation that scrolls from the right to left direction.

*Ramp Test.* The data set utilised for the “Ramp test” was similar to the one of the “step test,” but this time changing a single parameter, i.e., the number of frames. The test was conducted with 44, 31, and 21 frames, representing normal, brisk, and running human speeds. A ramp is a temperature perturbation that consists of a diagonal vector. A representative illustration of the “Ramp test” with six frames, and the ramp (shown in red) moving inside the 8 × 8 array from right to left direction, is shown in Figure 10.

**Figure 11:**
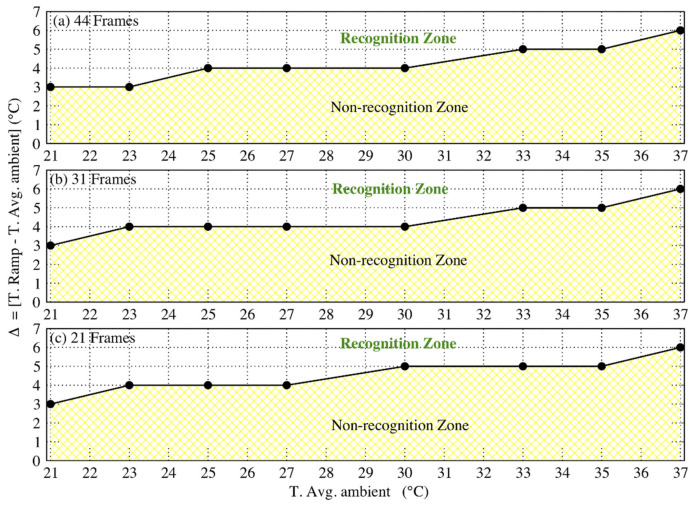
Plot of delta (Δ) Vs Average ambient temperature (T. Avg. ambient): (**a**) normal pace, 44 frames; (**b**) brisk pace, 31 frames; (**c**) running pace, 21 frames. The recognition and non-recognition zones are shown for each of these cases.

From the obtained results, we could establish that the limitations of the algorithm are strongly related to the average values of the ambient temperature and to the perturbation values. More precisely, we calculated delta (Δ) that represents the difference in temperature between the ramp and the average ambient condition. In Figure 11, we present the graph describing the variation of Δ as a function of the average ambient temperature for three different frame numbers. It is evident from Figure 11 that the algorithm can detect an event only for values greater than the minimum Δ value, specific to the average ambient temperature value. For instance, considering the event with 44 frames (Figure 11a), an average ambient temperature of 27 °C, and a ramp value of 34 °C, the value of Δ is then 6 °C, which is greater than the minimum Δ value (4 °C) required for recognition. Hence, this event is detected by the algorithm.

### 5.3. The Random Test

**Figure 12:**
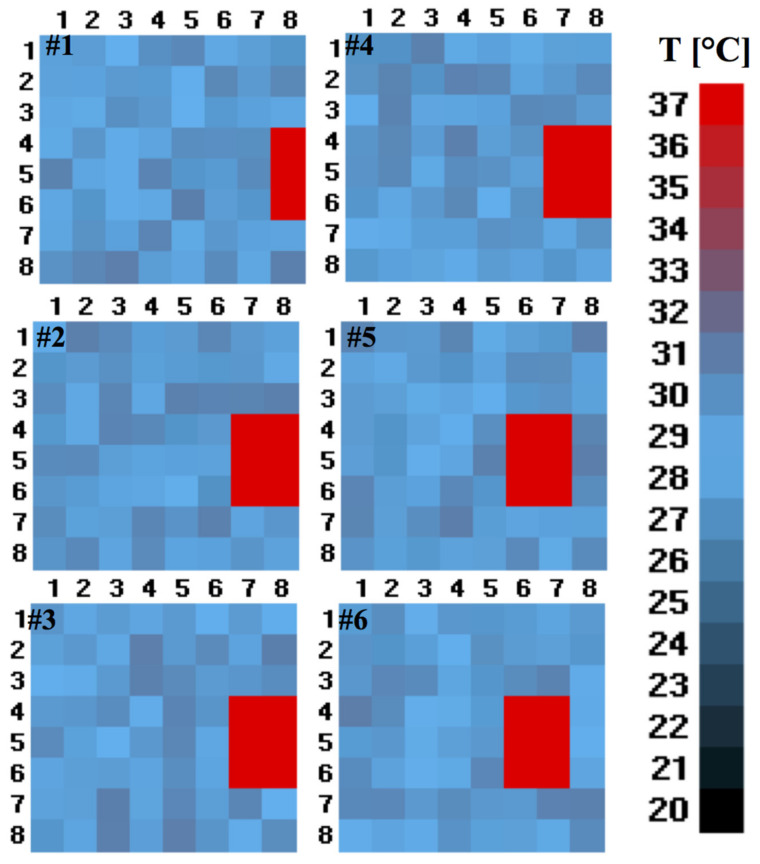
The representative illustration of the random test is shown here. The scenario chosen is when the person is at distance of 5 meters from the sensor and for 26–30 °C temperature range.

Our objective here was to simulate a realistic human passage and finally to assess the algorithm in a natural environment. To do so, we performed a random uniform distribution test by choosing the number of frames representing the normal (44 frames), brisk (31 frames), and running (21 frames) pace of a person. In detail, simulating the normal walking speed consisted of 5 empty initial frames (representing no passage), 34 frames with the passage at a set temperature and distance, and empty 5 terminal frames, in total 44 frames (5 + 34 + 5). In a similar way, the frames corresponding to a brisk (31 frames = 3 + 25 + 3) and a running pace (21 frames = 2 + 17 + 2) were chosen. The simulation of human presence consists of an image of 9 pixels (3 × 3), 6 pixels (3 × 2), and 2 pixels (2 × 1) at a temperature of 37 °C (some examples of these images are the red rectangles in Figure 12), representing, respectively, a distance of approximately 1.5 m (9 pixels, 9P), 5 m (6 pixels, 6P), and 7.5 m (2 pixels, 2P) between the sensor and the person crossing the passage.

The peculiarity of this type of test is that the frames were created using synthetic images, and the temperature values followed a uniform distribution within a precise temperature range. The selected ranges were: 18–22 °C, 22–26 °C, 26–30 °C, 30–34 °C, and 30–38 °C, typical temperatures present inside residential buildings with and without air conditioning systems. All these tests were repeated ten times and with four different user movement patterns: Input (I), Output (O), Input-Hold-Output (IHO), and Output-Hold-Input (OHI). In Figure 12, an illustration of a person at 5 m from the sensor (represented with 6 pixels) is shown. The different colour shades of pixels correspond to different temperature values.

**Figure 13:**
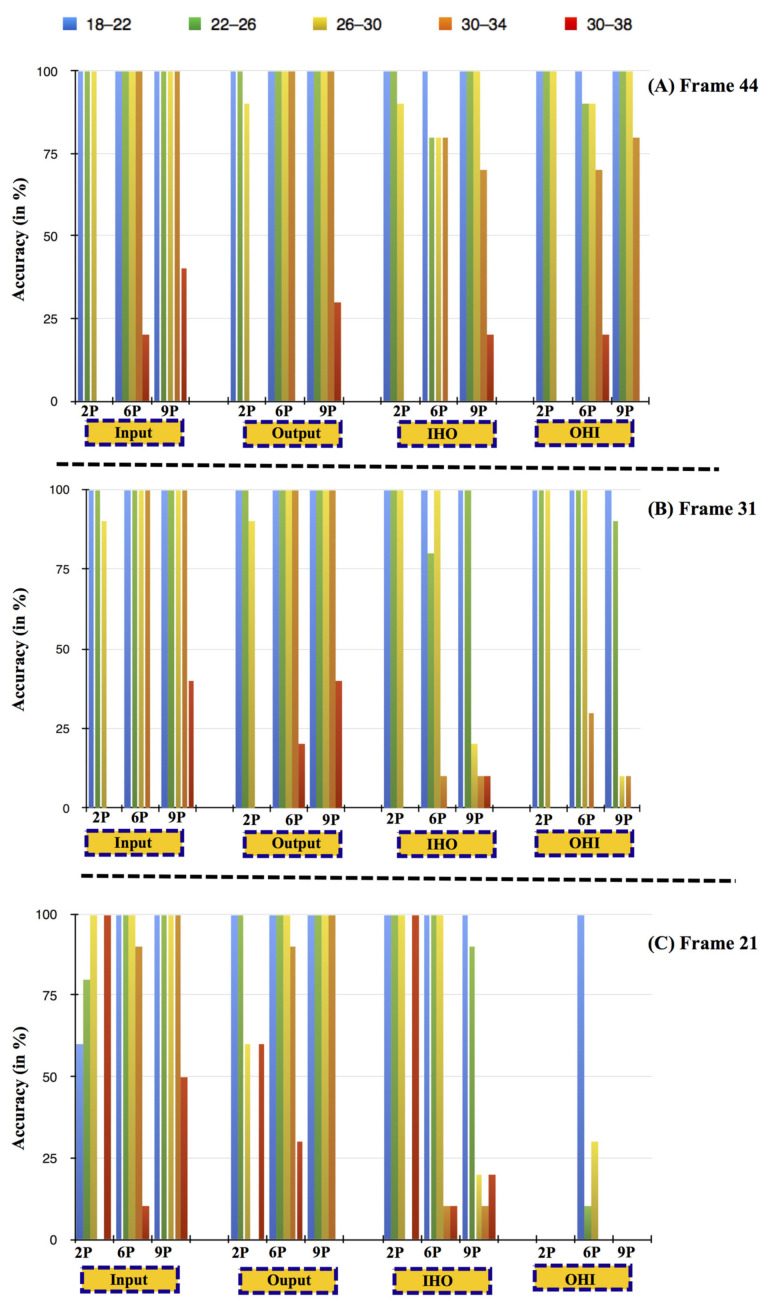
Accuracy plot for detection of four distinct human passage movement (Input, Output, IHO, OHI), at different distances (9P: 1.5 m, 6P: 5 m, and 2P: 7.5 m), and different temperature ranges (18–22 °C, 22–26 °C, 26–30 °C, 30–34 °C, and 30–38 °C); (**A**) 44 frames—normal, (**B**) 31 frames—brisk, and (**C**) 21 frames—running pace.

Figure 13 shows the algorithm’s accuracy considering four distinct human passage movement for the selected temperature ranges.

For an average walking speed (44 Frames, Figure 13a), independent of the distance from the sensor, the average detection accuracy of the algorithm is 95% (considering all four movements and for the ambient temperature ranges up to 30 °C). However, at higher temperature ranges (30–34 and 30–38 °C), the accuracy of the algorithm gradually decreases with the human distance from the sensor.

For brisk walking speed (31 Frames, Figure 13b), the algorithm’s accuracy is 90% or more for the ambient temperature up to 26 °C, independent of the number of pixels. If we consider only two main movements (Input, Output), the accuracy is 90% or more up to 30 °C ambient conditions. Moreover, for high-temperature ranges (30–34 and 30–38 °C), no detection (0% algorithm accuracy) of movements at approximate distances of 7.5 m from the sensor was observed, similar to the case of 44 Frames (average walking speed).

For running pace (21 Frames, Figure 13c), considering only the two main movements (Input, Output), the accuracy of the algorithm is 60% or more up to an ambient temperature range of 30 °C. The performance of the algorithm accuracy decreases as the human distance from the sensor increases. At a ~7.5 m distance from the sensor and at 30–34 °C, the algorithm was insensitive to any movement. 

In summary, the accuracy of the algorithm was found to decrease in the three cases:

- With an increase in walking speed. Given the fixed and small number of samples per second captured by the sensor, high walking speed will reduce the number of sensor elements activated and from the algorithmic point of view, the information available for discriminating between variations due to the passage under the gate or variations due to environmental thermal noise.
- With an increase in the user’s distance from the sensor. Given the fixed resolution of the sensor, if the subject is in the proximity of the sensor, more sensor elements will be activated in comparison with a subject far from the sensor that will have an impact on fewer sensor elements. Hence, if the user is far from the sensor, then the algorithm has less information for discriminating between variations due to the presence of a user under the gate or variations due to environmental thermal noise.
- At temperature ranges above 30 °C. As the ambient temperature increases becoming comparable with user temperature, the algorithm has less information for discriminating between variations due to the presence of a user under the gate or variations due to environmental thermal noise. However, considering that the application targets indoor monitoring, a normal thermal condition should satisfy thermal people’s comfort, i.e., indoor temperature lower than body temperature.

## 6. Conclusions

In this paper, we presented an approach that detects human presence in an indoor environment by combining thermal imaging provided by the IR sensor array with a novel pattern recognition algorithm. The IR sensor was implemented using the Z-Uno developers board, which allowed realizing a simple, low-cost sensory system by exploiting the potential of a Z-Wave communication protocol. The pattern recognition algorithm was able to detect passage with high accuracy of the passage of users with different walking speeds, at different user distances from the sensor, and temperature ranges typical of a residential environment. In particular, for indoor temperatures ranging between 21 °C and 31 °C, the accuracy of the proposed algorithm is 100% if the sensor elements activated by people at average walking speed passage measure a temperature higher than 35 °C. In general, the different distances between the sensor and the people passage area, different speeds of the people passages, and thermal noise can reduce the performance of the proposed algorithm. In fact, considering different walking speeds and an indoor ambient temperature that reaches up to 30 °C, the average detection accuracy of the algorithm decreased to 95%.

Future work will address the measurement of instantaneous power and electric energy consumption of the device, dimensioning battery capacities, and analysing energy harvesting technology, such as solar cells.

## Figures and Tables

**Table 1:** A comparison between 8 × 8 pixels thermopile IR sensors array methods reported in literature for human detection.

Reference	Case Study	Sensor Type	Feature Extraction Method	Accuracy
Proposed	Indoor people counter	IR array	Unsupervised pattern recognition based on moving average thresholding	95%
[16]	Tracking motion and Proxemics	IR array	K-means clustering	80%
[17]	People occupancy	IR array	Otsu’s Binarization and temperature filtering technique	93%
[18]	Indoor human detection	IR array	Activity recognition algorithm and Kalman filter for noise removal	70–95%
[19]	Indoor human detection	IR array	A probabilistic method with multiple pre- and post-image processing techniques	
[20]	Human localization and tracking	IR array	Adaptive threshold, Nearest neighbour, and Kalman filter tracking	-
[21]	Doorway Occupancy counter	IR array	Kalman filter tracking	89–92%.
[22]	Quantifying toilet usage in offices	IR array	Machine learning methods (KNN, SVM, LR, LSTM)	>98–99%

**Table 2:** Principal features of an IR sensor set-up investigated in this work [25].

Dimensions	11.6 mm × 4.3 mm × 8.0 mm (L × H × W)
Operating voltage	3.3 V or 5.0 V
Current consumption	Typ. 4.5 mA (Normal mode); 0.8 mA (Stand-by mode), 0.2 mA (Sleep mode)
Temperatures range of measuring object	With amplification factor High gain: 0 °C up to 80 °C, gain of the Low: −20 °C up to 100 °C
Field of view:	60° (vertical and horizontal)
Number of Thermopiles:	64 (horizontal × vertical 8 × 8)
Frame rate	10 frames/s or 1 frame/s
Absolute temperature accuracy	Typ. ± 2.5 °C

**Table 3:** The description of various events: actions performed by the users.

Identifier	Description
I: Input	People crossing (entering) the doorway
O: Output	People crossing (exiting) the doorway
IHO: I—Input H—Hold O—Output	People not completing a full doorway crossing (fake entry). The person comes from the entrance side, holds in the doorway and then goes back from the entrance.
OHI: O—Output H—Hold I—Input	People not completing a full doorway crossing (fake exit). The person comes from the exiting side, holds in the doorway and goes back towards the exit.
IHI: I—Input H—Hold I—Input	People completing a full doorway crossing with a hold. The person comes from the entrance side, holds in the doorway, and completes the crossing.
OHO: O—Output H—Hold O—Output	People completing a full doorway crossing with a hold. The person comes from the exiting side, holds in the doorway, and completes the crossing.

**Table 4:** Matching between time series and events. The ellipsis represents any series of values between 1 and 3 (for IHO and IHI) or between 2 and 4 (for OHI and OHO).

Time Series	Identifier
4321	I
1234	O
432…234	IHO
123…321	OHI
432…321	IHI
123…234	OHO

**Table 5:** The pattern of actions performed by the users. (For the acronyms of events, see Table 3).

N.	1	2	3	4	5	6	7	8	9	10	11	12	13
Event	I	O	I	O	IHO	I	OHO	IHI	I	O	OHO	I	OHI

**Table 6:** User ID, height, and total time for performing the actions described in Table 5. The total time is computed cumulating the crossing time at the base of the cone corresponding to the angular aperture of the sensor (Figure 7).

User	Height (cm)	Total Time (s)
U1	173	111
U2	175	101
U3	182	102
U4	170	93
